# Lessons from a Surgical Center Satellite Warehouse in a Large Brazilian Public Hospital

**DOI:** 10.3390/healthcare9030297

**Published:** 2021-03-08

**Authors:** Augusto da Cunha Reis, Renata Pereira Oliveira, Letícia Ali Figueiredo Ferreira, Cristina Gomes de Souza

**Affiliations:** Federal Center for Technological Education Celso Suckow da Fonseca, Rio de Janeiro 20271-110, Brazil; renatapoliveira@gmail.com (R.P.O.); leticialifig@gmail.com (L.A.F.F.); cristina.souza@cefet-rj.br (C.G.d.S.)

**Keywords:** healthcare, public health, developing countries

## Abstract

Brazilian public hospitals face several operational problems not only related to poor public management practices and their complex nature, but also the economic, and social contexts. Considering this scenario and the fact that efforts in supply management might affect a hospital organization’s excellence, this research aims to identify improvements in the logistic operations at the surgical center satellite warehouse of a Public Hospital located in Brazil. A case study based on an exploratory and qualitative approach was conducted by employing document analysis, semi-structured interviews, and on-site observations. Seven major problems concerning lack of surgical material, the non-definition of crucial logistic parameters, low information flow, surgical supply control, and management problems were pointed and addressed by seven independent but complementary actions that considered the Brazilian healthcare system’s particularities. Given the nature of exploratory research, the results are not exhaustive and cannot be generalized to different contexts. However, they help understand that reducing the waste of the logistics processes makes it possible to improve the attention to the local population that uses public health services.

## 1. Introduction

Based on the principle of health as a right for the State’s citizen and responsibility for the State, the Brazilian Constitution of 1988 instituted the Brazilian Unified Health System (or *Sistema Único de Saúde*–SUS), that it has intended to improve primary and emergency care [1]. However, Brazil continues facing several health-related problems caused by its new demographic profile and the primary challenges experienced by undeveloped countries [2]. Thus, Brazilians’ expectations and dissatisfaction with healthcare services have been growing and becoming a more recurring problem [3].

After the turn of the 21st century, the Brazilian population has grown and aged more prominently. According to the World Bank Group, it went from 174 million in 2000 to over 211 million in 2019, presenting a growth rate of 17% [4]. In that same period, the population share above 65 years more than doubled in size, reaching 19 million people [5]. These social changes combined jeopardize local health infrastructures and generate capacity and demand imbalances, especially in elective surgeries and emergency procedures performed in hospitals [6].

In addition, the Brazilian population suffers from several diseases caused and stressed by primary and fundamental issues, intensified by tropical nature [7], which helps to congest Brazilian hospitals [8,9]. In underdeveloped or developing countries, the current economic crisis also increases the difficulties faced by public hospitals that depend on a pre-determined budget and are thereby dealing with funding restrictions, which impacts the investment required to purchase material for surgery [10]. Additionally, there are problems concerning poor administrative management, lack of dignified attention to patients, and quality of service provided [11].

Health systems, especially hospitals are multifaceted and expensive structures as they provide healthcare and clinic assistance by managing several resources (human or material) within the same space, simultaneously [12]. Hospital management is invariably complex due to the challenging aspects of management, regulation, financing and technologies available [13].

Logistical distribution in hospitals must follow a consolidated strategy based on the variety of patient input, materials, and information. The increase in productivity of the existing system incorporated to the increase in logistical efficiency is considered the best alternative to improving the expectations of its users. From this point of view, hospital logistics deserves to be defined and emphasized separately, especially hospital materials [14]. In particular, efforts related to materials, stocks, and especially supplies’ logistics might affect the development of healthcare activities and the operational excellence of a hospital organization [15], making warehouses and storages play an important in the results [16].

In the hospital environment, the surgical center is one of the most complex units, given its logistical distribution, countless equipment, and type of assistance provided. Besides, many hospital processes and subprocesses are directly and indirectly associated with the surgeries [17]. Therefore, considering this scenario, this research aims to propose improvements in the logistics operations at the surgical center satellite warehouse of a Public Hospital located in Rio de Janeiro, Brazil, in order to avoid material absence—a recurrent problem. To achieve this goal, a case study based on an exploratory and qualitative approach was conducted.

The proposed practices seek to reduce surgical material waste and improve management. They might be a foundation for future works and a framework for implementing the suggested improvement actions in public hospitals in Brazil or other similar countries [18]. Besides, the results might help the local population by increasing the quality of the government’s service. As the literature on this subject is scarce, this study also contributes to hospital managers and researchers interested in this area.

## 2. Materials and Methods

This section describes the procedures used to prepare the case study, considering a qualitative and investigative approach [19]. The case study research has gained considerable acceptance as a research method all encompassing, and constituting a challenging endeavor that hinges upon the researcher’s skills and expertise through methods and practice [20]. In this paper, the case study methodology used was three-folded data:

In the first step, the healthcare sector was selected based on the precariousness of the Brazilian public healthcare sector and the need to both reducing costs regarding current government budget restrictions and improving health attention to the most deprived population [11,20,21]. Specifically the public sector, which is guaranteed by social and economic policies aimed at reducing the risk of disease and other diseases and universal equal access to actions and services [1]. The health is one of the main engines for a country’s development and economic growth, since it directly affects and can improve worker’s efficiency and, therefore, their productivity [21]. Thus, improvements in healthcare indicators might, in the long term, increase the gross domestic product (GDP).

Therefore, a large-sized federal public hospital located in Rio de Janeiro (Brazil) was selected in step 2. This hospital was chosen because it has a cooperation agreement term with the authors’ university, which illustrated the interest of its high administration for academic-based solutions. Besides, ease of access, the hospital’s interest in improving its care, and employees’ availability and cooperation were considered.

Finally, for the third step, data were collected from hospital documentation, on-site observation, and semi-structured interviews conducted with employees at all business levels. The hospital studied provided reports, warehouse ordering forms, processes’ maps, procedures reports, and manuals for analysis. For the semi-structured interviews, the researchers elaborated a set of questions directed to each interviewee. However, the interviews were conducted as an informal conversation, and the researcher had the flexibility to ask additional questions that had helped not only to elucidate unclear issues but also to redirect the interview when needed [22,23]. This type of interview has a high response rate as people are more accepting of personal interviews [24]. All the information collected were compared and compiled to elaborate on the case study.

The interviews were conducted with professionals from three hierarchical levels: strategic, tactical, and operational. Thus, from the strategic level, the hospital director and the head nurse from the surgical center were interviewed, and the questionnaire sought to obtain an overview of the hospital and its satellite warehouse. From the tactical level, it sought to obtain technical details about the warehouse operation, its integration with the administrative and supply area. Thus, the supply area coordinator and head of the warehouse were interviewed. In the operational area, the questionnaire was directed to the warehouse managers and administrative technicians, and it sought to broadly understand the operations, collecting information on difficulties and opportunities for improvement perceived by the employees. Although all the involved parties in the satellite warehouse operation were not interviewed due to limited research time, the chosen professionals allowed an overview of the situation.

In addition to the interview, the on-site observation allowed the researcher to acquire data and information about the hospital and the processes that would not be possible or enough of the perceiving directly. This technique helped the researcher to obtain information about certain aspects of reality and to identify and gather implicit or unaware information and evidence [25].

Along with these techniques, the best practices found in the academic literature, were used to perform a situational diagnosis that identified opportunities for improvement. Articles from the Science Direct, Emerald, Scopus, and Web of Science databases were retrieved, as they collect several scientific journals that adequately represent the field of study of interest. Literature findings were also used as a basis for the proposed solutions, and their possible benefits were described.

The methodologic summary of this paper is presented in Table 1. 

## 3. Empirical Study

### 3.1. Hospital Profile

The hospital selected for this study, is a Federal public tertiary hospital associated with the Brazilian Ministry of Health and located in the North Area of Rio de Janeiro, Brazil. It is a medium and high complexity unit founded in 1945. Its mission is to provide quality and humanized care and continuing professional education according to the SUS’s principles. For many years, this hospital has specialized in burn treatments, becoming a current reference in this area. It is also a reference in medium and high complexity cases and cancer treatment.

In addition, it is an open-door hospital, that is, it operates 24 h a day, 7 days a week. It works with elective surgeries, previously scheduled, and emergency surgeries from the primary network, in particular, due to chronic problems that worsened and the unfavorable environment. For sake of clarity and simplification, the hospital studied will be referenced as PFH, which means Public Federal Hospital.

According to Data Rio, the North Area, where the hospital is located, is the most extent area of Rio de Janeiro as it begins at a middle class residential and commercial zone and sprawls to the district known as *Baixada Fluminense* [26]. This area has about 33 slums and concentrates the most underprivileged share of the population. The specific district has 3.1% of its households below the poverty line and six slums surround it. Thus, the region suffers from problems related to poor or no proper sanitation and underdeveloped life conditions [27].

These demographic characteristics create a scenario of high inpatient flow and large demand due to the hospital’s great coverage area. Also, to address the surrounding population, the unit has one of the largest emergency services in the city, which is prepared to receive severe cases. As the hospital is located near one of the main city highways, it regularly receives multiple trauma cases caused by car accidents. Moreover, police operations in the slums and street violence are related to a high number of gunshots wound cases taken over by this unit. There is also a demand for stroke cases and diseases related to or aggravated by insalubrious or unhealthy environmental conditions [28]. Thus, what is observed is that these specificities not only increase the demand but also increase its unpredictability since a lot of the emergency cases attended by the hospital are due to unpredictable external factors. This impacts because patients are received at the hospital emergency room and referred to the surgical center. The materials that will be used in this surgery were not planned in the surgical map. It directly impacts the operation of the warehouse that is the object of study of this article.

### 3.2. Systematic Operation of the Satellite Warehouse

In the structure of the PFH, there are two different warehouses in the hospital: The first one, called Pharmaceutical Warehouse (PW), is the pharmacy, which is responsible for storing drugs, vaccines, and serums; the second warehouse stores common materials, such as diapers, syringes, gloves, and surgical supplies. It also stores specialized clinical materials called Orthotics, Prosthetics, and Special Materials (OPSM) [29]. Thus, it is called Material Warehouse (MW).

The MW is constituted by a central and a satellite warehouse. The central warehouse (CW) is located on the 2nd floor of the hospital and employs 22 workers. The satellite warehouse (SW), which is the object of this study, operates from inside the hospital’s Surgical Center, which holds 9 operation rooms and is placed on the 11th floor of the PFH. Also, it is run by 8 employees, operates in a 24/7 regime, and receives its supplies from the central warehouse.

Even though the PFH only has these warehouses in its official structure, it is common knowledge that, usually, some of its clinics have informal warehouses that store basic consumption materials for later use. According to Volland et al., it is customary for employees to maintain hidden supplies in hospital units, due to difficulties in implementing more organized structures [30]. For instance, there is a physical space within the hospital’s ambulatory to store and distribute supplies. This “informal” warehouse and distribution center is not an official part of the hospital, and it is run and maintained by the ambulatory employees to save time and assure material availability.

For storing material in the satellite warehouse, the OPSM materials, which are used mainly by the hospital clinics, are physically separated from the basic materials. The OPSM materials need more attention in their storage since they involve a high-cost flow and their inadequate management can directly affect the hospital financial outcomes [31].

Then, the raw material are all identified by their ID numbers and inserted in the HOSPUB and E-SUS systems. The HOSPUB is a public domain integrated computerized hospitalization system developed by the SUS Computer Department (DATASUS) and Ministry of Health. The E-SUS is a new hospital management system also developed by the DATASUS to replace HOSPUB and restructure its information.

Besides using both database systems, the hospital uses Excel spreadsheets to manage and control the materials stored. However, different forms are used for raw and OPSM materials. Thus, the raw material form requests the material ID, quantity, and withdrawal date as output information. Another form receives information regarding the OPSM materials. For this model, the ID, the quantity of material, the patient’s medical history, the number of issues, and the responsible employee are required. These data allow better material control and report generation as the name of the patient and clinic to which the OPSM materials were destined are recorded.

The material request process from the central to the satellite warehouse works as the following: the employee currently responsible goes through the shelves to check whether a certain material is available to be taken out. If no, they estimate the volume of items needed. Sometimes, this estimation is based on know-how. Other times, the quantity is estimated based on the number of items needed in the previous day. Raw materials are requested daily from the central warehouse. On the other hand, OPSM materials are requested according to surgical demand.

By using this daily and on-demand request process, there will be no chance of material lack in the operating rooms. However, according to the responsible head nurse and the hospital’s director, the lack of material is recurrent, and that often this material is available in the CW, but it is not sent to the SW. The hospital’s director pointed out during his interview that he is aware that there is a clear delay in the process of sending material from the CW to the SW, which causes the absence of supplies for some procedures at the time of use. For the head nurse, the warehouse is responsible for this problem as the nursing team procedures for requesting supplies are properly performed.

Another process observed during on-site visits was the assembling of basic materials kits for the clinics. These kits contain the materials required by the nursing team responsible for the surgeries and are always assembled on the previous day of the procedure. Thus, the satellite warehouse employees assemble kits that can attend an average of 4 surgeries daily. Even though the kit assembling is perceived as a good practice, it still does not assure the traceability of the material used in each procedure, as reported by the head of the warehouse. This interviewee also said that ideally tailored kits should be assembled for patient, as is usually done in private hospitals. As the kits are assembled for a type of surgeries, but it is not addressed for which surgery, it is not possible to identify which patient received the material.

Also, the employees do rounds to better communicate between sectors. These rounds are routines in which a multidisciplinary team from each clinic, on a specific day of the week, evaluates the clinical status of each patient. These rounds support the surgical map, except for emergency surgeries. By using the map, the warehouse determines the demand for inputs and programs itself in terms of next-day supply. This program includes the assembling of surgical kits, for instance.

### 3.3. Gaps of the Satellite Warehouse Operation

The new hospital management system (E-SUS) that was installed to replace the previous one (HOSPUB) was not fully implemented due to lack of investment and poor management. Therefore, to maintain the information record, the hospital uses these two databases and the Excel forms simultaneously, which generates rework, cross information, and the possibility of failures as the systems do not communicate with each other. Besides, due to difficulties in material management and control, some surgeries are scheduled without previously verifying the storage availability of OPSM materials. This impacts the expectations of patients who end up frustrated when scheduled surgeries do not occur.

There is not a structured material ordering process for the satellite warehouse studied. The material availability is manually checked and depends on know-how. When previous demand is used, there is no implementation of any validated method, and only the number of items used in the previous day is consulted. Therefore, the warehouse faces a constant shortage of supplies. Also, it is noteworthy that there is neither the use of a safety stock nor planning strategies to avoid problems in the ordering material process. When it comes to inventory management, there is also little control over the actual use of the basic materials. It is not possible to track in which clinic or patient the materials were used because its specific form does not request any information of this sort. Thus, basic material reports do not present any entry for doctors, patients, or clinic identification.

In Brazilian public institutions, the acquisition of goods and services is a complex process. Very extensive steps constitute the bidding law (Law 8.666/93), and it is incredibly bureaucratic and not very flexible [32]. Moreover, there are budget-related problems, supplier relationship-related problems, and delivery problems that may also result in material shortage.

Another point that contributes to this is the assembly of kits for clinics. The kits of basic materials assembled for the clinics do not allow any traceability of the material used in each patient. In contrast, it is very difficult to assemble a specific kit since each patient has a procedure and a list of materials. Therefore, it was raised the possibility of pre-assembled kits that would contain the materials regularly used by the clinics during surgical procedures.

There is also a need to improve the material flow inside the hospital. Sometimes, some materials are requested for a surgery or procedure but are not used. Thus, it should be possible to return them properly to the warehouse. As there are no periodic meetings between the surgical center and the satellite warehouse team, this and other problems are not addressed.

The surgical map is available for the surgical center and the satellite warehouse teams in 24-h advance. Elaborating this map is an important source of communication and can help plan the kits, but there are still problems. The main one is related to the frequency of the rounds. As they happen only once a week, the surgeries that are planned or scheduled after that are included in the surgical map late, which can cause organization problems.

Finally, it was perceived a lack of employees’ training, which directly affects communication and information flow processes. There is a high turnover rate, and many of the workers are outsourced and present limited knowledge of the processes.

## 4. Improvement Proposals

According to Aguilar-Escobar et al., between 30% and 40% of the total hospital expenses are invested in logistics activities, considered as a potential improvement objective to increase efficiency in budget management [33]. Thus, measures regarding its improvement are suggested below.

To improve system communication and assure better information and data flow, HOSPUB discontinuity is suggested. In this case, only the e-SUS system would keep running. According to DATASUS, the e-SUS objective is to facilitate and contribute to the organization of health professionals’ work. The e-SUS updates keep the system in perfect working conditions and must be required by the PFH strategic level to the Ministry of Health to ensure data transparency and avoid information loss. Also, since e-SUS maintains electronic medical records recording patients’ evolution, medical prescriptions, among others, it can help in the communication between the surgical center and the satellite warehouse [34].

To reduce OPSM material shortage in surgical procedures, the request for this type of material from elective procedures, according to the Ministry of Health, must be made at least 48 (forty-eight) hours in advance, using a specific form from the data system, which must include the patient’s name, the medical record number, and the name of the health professional responsible. This measure would give enough time to transfer the patient to another hospital or reschedule their surgical procedure in case there is a shortage of a needed surgical supply [35].

Another proposition is that surgeries should only be scheduled following OPSM materials availability verification. It would assure that the material delivered for the procedure is correct and meets the needed requirements. This measure addresses the problem related to the constant cancellation or rescheduling of surgeries, which not only frustrate and compromises the patient’s care but generates rework and process delays.

The shortage of material is a regular problem in the satellite warehouse of the surgical center. A study indicates that although it can be associated with financial issues, the lack of general supply in the CW and system failures are also responsible for the material shortage in the SW [33]. Thus, modifying the material ordering process, define the reorder point, and maintain good supplier-client relationships are essential to minimize these problems. Also, the reorder point and the delivery time must follow what is established by the bidding law, which is followed by public hospitals.

According to Paschoal and Castilho, the reorder point corresponds to the number of items that trigger an action to replenish a particular inventory [36]. This number is related to the safety stock, which is the minimum stock necessary for the continuity of the activities until new supplies are received. The undefinition of this reorder point is directly related to a lack of material problems, as it is an important parameter for stock control. Works of Lapierre and Ruiz and Pan and Pokharel indicate the need to coordinate this reorder point, allowing scheduled deliveries, respecting availability, and avoiding large stock levels [37,38]. The basic rule to calculate the reorder point is to know the average demand, lead time, and minimum stock [17].

Su et al. suggest, as a best practice, a weekly order system for basic materials, in which all the request orders are filled in a single weekday [39]. The material check might take place the day before to cushion the stock and avoid a possible supply shortage. This measure would increase storage space, improve inventory management control, and reduce staff time, and waste. On the other hand, for the OPSM request, studies suggest that the best process is to keep a minimum stock level in the satellite warehouse and request materials according to the elective surgery schedule. Although hospitals provide essential services and require above-average inventory levels, this format goes according to the just-in-time methodology, which seeks to operate with minimum inventory levels and maximum rationalization of resources [37,40,41,42].

In some cases, the delivery delay also causes a lack of stored material on both warehouses. Then, an excellent supplier-client relationship is recommended. Pre-established contracts are proposed to ease these processes [30,43]. In his study, law highlights the importance of this partnership to improve delivery performance [44].

Another improvement point perceived is to assemble kits for all surgical center procedures. Van de Klundert et al. suggest the need to have a specific kit for each type of surgery, one for all surgeries and a hybrid kit [45].

Currently, the satellite warehouse of the PFH does not control or keep track of the raw materials used in the surgical centers. Doing so would improve the demand forecasting as information about the type of surgery to which the supplies were requested would be known. It would also help to estimate the financial expenses related to materials ordering by specialty. Therefore, it is suggested that such as the forms of OPSM materials, the inventory control form for raw materials should include the patient’s name, the type of the surgery, and the destination clinic.

Therefore, it is suggested that, upon receiving the surgical map, which should be done in daily rounds in all clinics, the kits are prepared per patient, indicating their respective name and corresponding medical history. It is also important that there are a basic kit and pre-assembled specific kits for all emergency rooms. This will help the nurses in managing time with their core activities. Bloss showed that 30% of nurse’s time is spent away from inpatient beds and patient care, as they work in activities that are not part of their core activity, such as the search for medications, supplies, test results, and other logistic activities [46].

Poksinska also highlights the importance of promoting and encouraging organizational learning, as well as aligning goals and involving everyone’s participation cooperatively [47]. Thus, fortnightly meetings between the warehouse manager and his team are proposed to receive feedback, map the problems, and brainstorm. Feedback and brainstorming practices can be seen in the work of Su et al., indicating the need to improve communication and share ideas stimulating everyone’s participation [39]. For improving communication between warehouse employees and the surgical center, activities that provide greater integration between sectors are proposed. The first suggestion consists of management meetings for work evaluation and forecast. Therefore, monthly meetings attended by processes leaders, are suggested to assist in process aligning and failures adjusting [47].

Finally, employee training is essential for the development of sectors and the progress of the information flow. Van Lent et al. indicate that although most hospitals support logistics education, few have permanent training programs, which impacts the development of team skills [48]. Therefore, training courses are proposed to be lectured in the hospital’s study center and public education institutions, through a partnership with the hospital.

Table 2 shows a summary of the main propositions of this work.

## 5. Conclusions

The demand unpredictability in the health supply chain, especially in hospitals, causes lack of stock, inefficient and expensive operational practices, as well as incomplete information flows [41]. This unpredictability is even more accentuated in public hospitals that operate with open doors and that have little government incentive to act on [49].

In this sense, this study aimed to analyze logistics operations at the surgical center satellite warehouse from a Brazilian reference federal public hospital. The focus was on proposals for solutions to improve operational efficiency in logistical processes in order to avoid material shortages, reduce waste, and optimize procedures. The solutions proposed might generate more financial and organizational outcomes, in addition to improving service to the local population, which is broadly used and proven effective in healthcare contexts [50,51,52].

During the case study preparation, it was noted the constant lack of storage material for elective surgeries, the non-definition of crucial logistic parameters (such as reorder point and minimum stock), poor communication, and surgical supply control, and management problems. All these factors combined hinder the demand and logistical management satellite warehouse to attend the surgeries of each clinic.

To improve the logistics processes of this warehouse, seven proposal actions were suggested based on best practices retrieved from papers found in the academic literature about healthcare [53]. Thus, all proposals are well-grounded and had already been proven effective when applied in similar contexts, taking into consideration its particularities [31,35,36,37,40,44,47,48].

Consequently, this work highlights the need and relevance of research that considers unfavorable contexts, such as the Brazilian one, to expand its possibilities for some practical application of advances and developments in the field of healthcare innovation. Moreover, as the analyses were conducted in the sphere of public management, it could also allow a better understanding of the particularities related to it, which includes public legislation, bureaucratic procedures, and government-established indicators. The improvements indicated for the PFH might provide process waste reduction, enhance the communication between the warehouse and the surgical center, and improve patient care and attention. Also, one hopes the same improvements suggested for the warehouse can be implemented in other hospital sectors and units.

Therefore, for future work, it is proposed the application of improvement actions suggested for the warehouse studied analyzing their real benefits with a quantitative approach. It is also suggested to continue to investigate the logistics processes in emerging or underdeveloped countries, considering its public management practices and the complexities of their public healthcare system to better understand their processes and the relevant tools to their specific context.

## Figures and Tables

**Table 1:** Methodological structure.

Step	Approach	Objective	Source	Aim
Diagnosis	Analytical	To select a hospital and identify possible improvements in its logistics processes.	Hospital documentations (reports, processes’ maps, and procedures and manuals)	To define the hospital structure and obtain information for this case study
			Semi-structured Interview: (i) Strategic level: hospital director and head nurse of the surgical center; (ii) Tactical level: supply area coordinator and head of the warehouse; (iii) Operational level: warehouse managers and administrative technicians	To understand the hospital and warehouse through employees’ perspective as well as to collect detailed information on difficulties, and opportunities for improvement perceived by these employees.
			On-site observation	To perceive and observe the actions of those involved in the warehouse processes and practical procedures.
Analysis	Theoretical and Analytical	To evaluation the theoretical and analytical data to prepare the case study.	Diagnosis	To search for literature-oriented solutions, establishing essential criteria so that the suggested improvements can be implemented in the hospital’s warehouse.
Proposition	Theoretical and Analytical	Preparing the case study and propose improvements in the processes.	Analysis	To increase the efficiency of the logistics processes of the hospital and reduce waste.

Note: The table shows the main steps followed by the author to conduct this study. Each step has a singular objective and is characterized by a specific method or technique for achieving its purpose. The column “Approach” indicates whether a step is based on documentation analysis or depends on practical actions. The column “Source” indicates what is the source of information to complete that step and what technique or method is employed to conduct the analysis. The “Aim” specifies what type of data and information were expected from that source.

**Table 2:** Methodological structure.

Problems	Improvements	Benefits
1. Material shortage for elective surgeries	(a) OPSM material must be requested in 48-h advance; (b) Surgery scheduling only after checking material availability; (c) Employment of a singular information system to improve material management.	Better organization of surgery, avoiding rescheduling and cancellations due to material shortage.
2. Material ordering process	(a) Definition of logistic parameters such as safety stock and reorder point considering lead time; (b) Weekly requests for basic materials.	Improve storage space, reduce waste, as well as improve control of received items.
3. Complexities in bidding processes and material delivery	(a) Maintenance of a good supplier-client relationship and establishment of pre-established contracts;	Streamline processes and improve delivery.
4. Assembling of basic material kits per clinic	(a) Basic and OPSM kits prepared per patient; (b) Basic kits and a pre-assembly of specific kits for all emergency clinics.	Allow material traceability and streamline internal surgical center processes
5. Absence of periodic meetings	(a) Monthly meetings between those responsible for the warehouse sector and the surgical center. (b) Biweekly meetings of the warehouse manager with his team, with the use of feedback and Brainstorming practices.	Align processes and adjust incongruences between sectors; Map the problems and explore the creativity of the group.
6. Surgical center rounds occur only once a week	(a) Daily rounds in all clinics.	Avoid information delay and facilitate the control material distribution.
7. Lack of training	(a) Offer training courses at the hospital’s study center and state public education institutions.	Develop team skills technical, operational, and management.

Note: This table summarizes the main problems identified in the warehouse. In the “Improvement” column, each problem is associated with one or more improvement actions and the column “Benefits” shows the main outcomes of the improvement actions.
